# The One That Got Away: How Macrophage-Derived IL-1β Escapes the Mycolactone-Dependent Sec61 Blockade in Buruli Ulcer

**DOI:** 10.3389/fimmu.2021.788146

**Published:** 2022-01-26

**Authors:** Belinda S. Hall, Louise Tzung-Harn Hsieh, Sandra Sacre, Rachel E. Simmonds

**Affiliations:** ^1^ Department of Microbial Sciences, School of Bioscience and Medicine, Faculty of Health and Medical Sciences, University of Surrey, Guildford, United Kingdom; ^2^ Brighton and Sussex Medical School, University of Sussex, Brighton, United Kingdom

**Keywords:** Buruli ulcer, *Mycobacterium ulcerans*, mycolactone, IL-1β, Sec61, protein translocation, macrophages

## Abstract

Buruli ulcer (BU), caused by *Mycobacterium ulcerans*, is a devastating necrotizing skin disease. Key to its pathogenesis is mycolactone, the exotoxin virulence factor that is both immunosuppressive and cytotoxic. The discovery that the essential Sec61 translocon is the major cellular target of mycolactone explains much of the disease pathology, including the immune blockade. Sec61 inhibition leads to a loss in production of nearly all cytokines from monocytes, macrophages, dendritic cells and T cells, as well as antigen presentation pathway proteins and costimulatory molecules. However, there has long been evidence that the immune system is not completely incapable of responding to *M. ulcerans* infection. In particular, IL-1β was recently shown to be present in BU lesions, and to be induced from *M. ulcerans*-exposed macrophages in a mycolactone-dependent manner. This has important implications for our understanding of BU, showing that mycolactone can act as the “second signal” for IL-1β production without inhibiting the pathways of unconventional secretion it uses for cellular release. In this Perspective article, we validate and discuss this recent advance, which is entirely in-line with our understanding of mycolactone’s inhibition of the Sec61 translocon. However, we also show that the IL-1 receptor, which uses the conventional secretory pathway, is sensitive to mycolactone blockade at Sec61. Hence, a more complete understanding of the mechanisms regulating IL-1β function in skin tissue, including the transient intra-macrophage stage of *M. ulcerans* infection, is urgently needed to uncover the double-edged sword of IL-1β in BU pathogenesis, treatment and wound healing.

## Introduction

Buruli ulcer (BU) is a neglected tropical disease resulting from subcutaneous infection by *Mycobacterium ulcerans*. It typically presents as painless, ulcerative skin lesions or as pre-ulcerative nodules, plaques and in oedematous forms (1). This environmentally acquired infection is reported in 33 countries world-wide, but is most common in West Africa and Australia (2, 3). The disease has a high morbidity, causing both disfigurement and disability (1). Untreated, the infection can become chronic and extend up to 15% of body surface-area. When diagnosed early, treatment with antibiotics alone can be effective (4). More severe cases may also require surgery, including debridement, skin grafts or, in extreme cases, amputation. Physiotherapy and long-term rehabilitation are frequently required where the disease has affected joints and even in high resource settings wound healing can take over a year (5).

### *M. ulcerans* and Mycolactone

In contrast to the granulomatous immune responses typical of mycobacterial infections such as TB and leprosy, BU lesions usually display clusters of extracellular bacilli within the necrotic tissue with a relative paucity of infiltrating leukocytes. This immunosuppression, affecting both innate and acquired immune responses, is due to mycolactone, the exotoxin virulence factor unique to *M. ulcerans* (6).

Mycolactone production is encoded by a large plasmid, acquired by *M. ulcerans* and the other mycolactone-producing mycobacteria during their evolutionary emergence from a common ancestor highly similar to *M. marinum* (7, 8). The chromosomal genome of *M. ulcerans* is ~98% identical to that of *M. marinum* (9), sharing more than 4,000 orthologous genes (10). However, extensive gene rearrangements, expansion of pseudogenes and frequent insertions of *IS2404* and *IS2606* have led to loss of several virulence factors. For example, *M. ulcerans* has lost two out of the five ESX secretion systems, including the ESX-1 and its substrate, ESAT-6, which facilitate mycobacterial phagosomal escape into the cytoplasm (9). It seems likely that these losses are compensated by the gain of mycolactone, since this diffusible, lipid-like molecule has wide ranging effects on host cells and tissues that replicate the pathological features of BU (6).

Mycolactone mimics the effects of *M. ulcerans* infection upon injection into tissue (6) inducing immunosuppression, analgaesia and endothelial cell dysfunction (11, 12). Mycolactone suppresses both innate and adaptive immune responses, ablating production of cytokines and chemokines by monocytes, macrophages, dendritic cells and T cells (13–19), preventing the induction of immune receptors and co-stimulatory proteins (13, 17), and restricting antigen presentation (13–16, 20). This wide-reaching immunosuppression is thought to be central to the lack of systemic inflammation seen in BU patients and explains the dearth of inflammatory cells close to the bacteria within lesions.

Furthermore, mycolactone is both cytopathic and cytotoxic, causing cytoskeletal rearrangements, rounding up and detachment of cells (6, 21), cell cycle arrest (6), disturbance of Ca^2+^ flux (17, 22), oxidative stress (23–25), activation of the integrated stress response (26, 27), autophagy (28, 29) and, eventually, apoptosis (26, 30). Importantly, in most cell types, apoptosis occurs at least several days after exposure *in vitro* (11, 26). In BU lesions there is strong evidence of mycolactone-induced apoptosis (31) and tissue necrosis, with debris from neutrophils and macrophages in close proximity to the bacteria (32–34). There is also cytotoxicity to neurons that may underpin the analgaesia (35, 36), although alternative explanations have also been proposed (37).

### Sec61 Inhibition by Mycolactone

**Figure 1:**
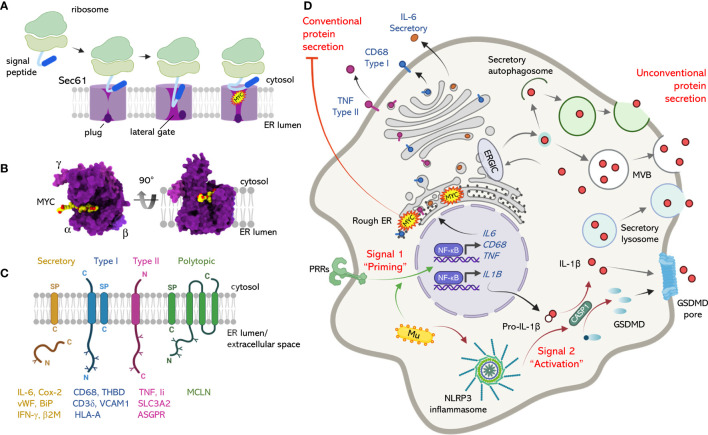
The mechanism of action of mycolactone at the Sec61 translocon and its influence over conventional and unconventional secretion. **(A)** Sec61-dependent co-translational translocation of proteins into the ER involves recognition of a signal peptide (SP) or signal anchor by the signal recognition particle and its receptor (not shown), which transfer it to Sec61. This results in reorganization of the translocon and movement the Sec61α plug domain, opening the central pore and allowing transit of the translating protein into the ER. Mycolactone binds Sec61α, preventing the SP from accessing its binding site at the lateral gate. Although the lateral gate is open, the plug remains closed and the translocon is locked in an inactive state. **(B)** The structure of inhibited Sec61, with mycolactone bound inside the lateral gate of Sec61α. Dark purple; Sec61α, light purple; Sec61β, pink; Sec61γ, yellow/red = mycolactone (from PDB:6Z3T). Two views are shown, looking down from the cytosol towards the ER and from the side, as in **(A)**. **(C)** Mycolactone-dependent inhibition of Sec61 affects a wide range of proteins, including secretory proteins with SP, type I transmembrane proteins (N-terminal ‘out’, with SP), type II transmembrane proteins (N-terminal ‘in’, with signal anchor), and a subset of polytopic proteins (particularly those with an SP). **(D)** Conventional and unconventional secretion of cytokines and inflammatory mediators. Stimulation of macrophage pattern recognition receptors (PRRs) promotes activation of signalling pathways (Signal 1) e.g., NF-κB, leading to transcriptional induction of many genes, including conventionally secreted proteins (eg IL-6), type I transmembrane proteins (eg CD68), type II transmembrane proteins (eg TNF, cleaved from the cell surface by ADAM17, not shown), as well as unconventionally secreted proteins (eg IL-1β). Here, the pathways diverge, with conventionally exported inflammatory mediators entering the ER *via* Sec61 while pro-IL-1β translation occurs in the cytosol. Activation of the NLRP3 inflammasome (Signal 2) activates pro-caspase 1, which cleaves pro-IL-1β to its mature form. Mature IL-1β is secreted *via* unconventional secretion pathways involving gasdermin D (GSDMD) pore formation, and/or membrane-bound organelles such as multivesicular bodies (MVB), autophagosomes and secretory vesicles loaded *via* TMED10 (not shown) at the ER-Golgi intermediate compartment (ERGIC). *M. ulcerans* (Mu) was recently shown to provide Signal 1 by means of TLR2 activators on its cells surface, and Signal 2 *via* mycolactone (MYC), which also inhibits Sec61 at the ER membrane. Figures generated using BioRender.com and Chimera X (https://www.rbvi.ucsf.edu/chimerax).

We discovered that mycolactone inhibits co-translational translocation of proteins into the endoplasmic reticulum (ER) by the Sec61 translocon (16) (**Figure 1**), and it is now clear that this mechanism underlies most of the biological actions of mycolactone laid out above. Crucially, this is the first mechanistic explanation for the biological effects of mycolactone that has been corroborated by multiple labs worldwide (16, 18, 38, 39), and is now accepted by the WHO as the mechanism that substantially explains the immunosuppression and cytotoxicity in BU (40).

It is useful to understand the details of Sec61 inhibition by mycolactone, as it applies across the nucleated cells of higher eukaryotic species that harbor *M. ulcerans* infections (41, 42). The Sec61 translocon consists of 3 subunits, Sec61α, Sec61β and Sec61γ, with Sec61α being the dynamic major pore forming subunit. It can open in two directions – *via* a central pore between the cytosol and ER, and sideways *via* a “lateral gate”. When the majority of secretory or membrane proteins are synthesized, they undergo a process of co-translational translocation. Here, their signal peptide or signal anchor engages with a specific site on Sec61α, which then opens both the lateral gate and the pore (**Figure 1A**). Mycolactone acts by binding to Sec61α (18, 43) and blocking the transit of proteins through the pore (38, 44). We recently resolved the structure of Sec61 bound to mycolactone using cryo-electron microscopy (43), which revealed that mycolactone occupies the entrance to the signal peptide binding site and, although the lateral gate is open, the channel remains closed (**Figure 1B**). Importantly, Sec61α is no longer able to move dynamically, and is instead trapped in this unproductive conformation.

This model fits well with the biochemical data, where mycolactone inhibits translocation of signal peptide-bearing proteins that use Sec61 to access the secretory pathway, i.e. co-translationally translocated secretory proteins, type I and type II transmembrane proteins, and a subset of polytopic proteins (18, 27, 44) (Hall, Hsieh, Simmonds et al., unpublished) (**Figures 1C, D**). Based on these features, and the high conservancy of Sec61-dependent translocation (45), the pattern of proteins affected by mycolactone in any given cell type is highly predictable. However, since constitutively expressed proteins are depleted at the turnover rate (11), which is highly variable, some proteins are lost more rapidly than others (27). On the other hand, induced (e.g. immune) responses are extremely sensitive to mycolactone’s effect on new protein production. Hence we and others (46) believe that many, if not all, of the cellular effects of mycolactone can be explained by Sec61 inhibition. Indeed, some observations previously ascribed to other mechanisms can retrospectively be ascribed to Sec61 inhibition, such as depletion of the type I transmembrane proteins e-Cadherin (21), L-selectin (20) and CD3 (17). Formal links have now been proven between Sec61 inhibition and many different aspects of mycolactone’s functions. These include loss of macrophage inflammatory mediators (16), T cell responses (18) and antigen presentation (47).

Translocation blockade is also intrinsically linked to mycolactone’s cytotoxicity, as forward genetic screening identified mutations in the gene for Sec61α, that reduce its ability to bind mycolactone (43). Cells carrying these resistance mutations survive and replicate in the presence of mycolactone (18, 26, 39, 43). Our working model is that the translocation blockade results in protein translation in the wrong cellular compartment (i.e. the cytoplasm), where they are degraded by the proteasome (16) or later removed by selective autophagy (28) (**Figure 1D**). Eventually these systems become overwhelmed, resulting in proteotoxic stress. Inhibition of Sec61 has been shown to be directly responsible for Ca^2+^ flux disturbance, induction of an integrated stress response and autophagy (22, 26, 28).

## Evidence for an Immune Response to *M. ulcerans*

Despite the fundamental process that mycolactone inhibits, and the critical role that secreted proteins and membrane receptors play in the immune response, there is significant evidence for an immune response to infection with *M. ulcerans*. For instance, despite reports of T cell anergy in BU patients, T lymphocyte responses to *M. ulcerans* antigens can be found in the blood of most BU patients (48–51). Notably, these responses can also be detected in uninfected people living in endemic areas (52, 53), even if they bear no sign of clinical disease, suggesting previous subclinical exposure to *M. ulcerans*. Furthermore, granulomas may develop in BU patients with late-stage disease (54) and spontaneous healing without antibiotics has also been reported (55–57). Hence, it is possible that many BU cases could eventually self-heal without medical intervention.

Control of *M. ulcerans* infection is reported in both guinea pig (58) and FVB/N mouse strain models (59). Initial evidence suggested the latter involves innate but not acquired immune responses (59) but recently, Foulon and colleagues demonstrated a correlation with the emergence of plasma cells within the local skin tissue, and mycolactone inhibitory antibodies (60), as well as down-regulation of mycolactone synthesis (61), although the drivers of this are unclear.

While these observations might be explained by the spatial distribution and local concentrations of mycolactone in infected tissue, genetic studies provide further evidence that macrophage responses are important in *M*. *ulcerans* infection. Many of the polymorphisms that affect either the likelihood of developing BU or disease severity impact the innate response to intracellular infection (62–65). For example, SNPs in the gene for the macrophage activating cytokine IFN-γ (*IFNG*), and *iNOS*, the inducible nitric oxide synthase that generates bactericidal NO in macrophages, both increase susceptibility to infection. Interestingly, SNPs in three genes involved in the autophagy pathway also affect BU. Two polymorphisms have been identified in the E3 ligase *PARK2* which increase susceptibility to BU while one in *NOD2* increases the risk of severe disease (62). By contrast, a SNP in *ATG16L1* is associated with reduced risk of ulceration (62, 65). This is particularly intriguing as we recently uncovered a protective role for autophagy in the cellular response to mycolactone (28).

**Figure 2:**
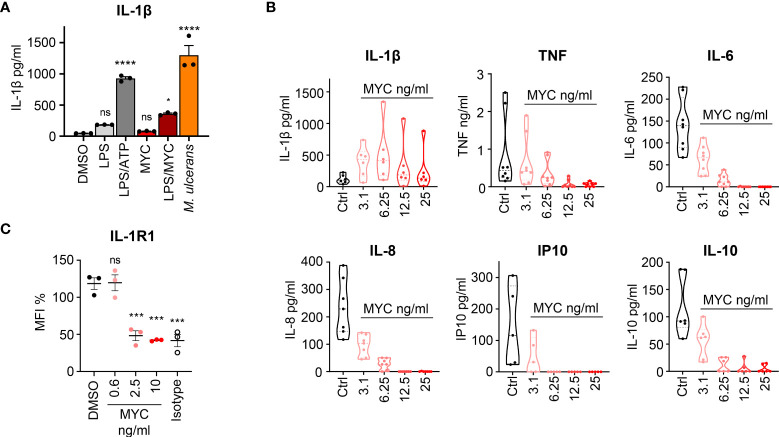
Induction of macrophage IL-1β activation and secretion by *M. ulcerans*. **(A)** IL-1β production by PMA-activated THP-1 cells incubated for 24 h with 0.05% DMSO, 10 ng/ml *E.Coli*-derived LPS (Enzo) with or without 1 mM ATP or 31.25 ng/ml synthetic mycolactone (MYC) or *M. ulcerans* Mu_1082. At this time point THP-1 cell viability was >95%. IL-1β was measured by ELISA (eBioscience) in triplicate (mean ± SD). Results are representative of duplicate experiments. **(B)** Rheumatoid arthritis (RA) synovial membrane cultures were isolated as in (67) and then cultured in medium alone or medium containing increasing concentrations of natural mycolactone (MYC) for 24 h. The concentration of IL-1β, TNF, IL-6, IL-8, IP-10 and IL-10 in the cell supernatants were measured by ELISA (68–70). This mixed cell population, including predominantly CD45^-^ fibroblast-like cells, CD14^+^CD45^+^ macrophages and CD3^+^CD4^+^ T lymphocytes, are highly activated, and spontaneously produce cytokines without further stimulation. In addition to those shown, these cells also secrete other cytokines/chemokines/inflammatory mediators including CCL5, CCL2, GM-CSF, IL-23, IL-17, IL1RA, IL-11,TNFRSF1B (TNFR2), MMP1, MMP2, MMP3, MMP13, and TIMP1, but not IFNγ or lymphotoxin (67, 71–76). All RA patients gave written informed consent and the study was approved by the Riverside Research Ethics Committee, REC number: 07/H0706/81. Violin plots showing median and quartile for n = 5-8 patients; median control values for the measured cytokines were IL-1β (103 pg/ml), TNF (433 pg/ml), IL-6 (139 pg/ml), IL-8 (229 pg/ml), IP-10 (116 pg/ml) and IL-10 (92 pg/ml). **(C)** IL-1R1 surface expression in human dermal microvascular endothelial cells exposed to 0.02% DMSO, or increasing concentrations of synthetic mycolactone (MYC) for 24 h. Cells were dissociated, stained with anti-IL-1R1 antibody (PA546930, Invitrogen) or isotype goat IgG (AB-108-C, bio-techne), donkey anti-goat IgG FITC (A16000, Invitrogen), and subjected to flow cytometry analysis. Mean fluorescence intensity (MFI) is presented as a % of untreated control (mean ± SEM, n = 3). For all panels ns; not significant, *P < 0.05, ***P < 0.001, ****P < 0.0001 using a one-way ANOVA and Dunnett’s multiple comparison test.

The most recent evidence of an ongoing innate immune response to *M. ulcerans* bacteria is the discovery that macrophages produce IL-1β following exposure to mycolactone (66). Given that most other cytokines and chemokines made by macrophages are Sec61-dependent and therefore strongly suppressed by mycolactone, this discovery likely has especial importance for BU. We have therefore validated this important finding in our laboratory (**Figure 2**), as will be discussed in more detail below.

## IL-1β

IL-1β, produced predominantly by monocytes and macrophages, is a potent pyrogen and one of the ‘alarm’ cytokines known to have widespread and systemic effects once induced (77). Foulon and colleagues detected IL-1β in lesions arising from *M. ulcerans* infection in both BU patients and in animal models. *In vitro*, IL-1β was induced in both murine and human primary macrophages, by a variety of different mycolactone-containing stimuli, including purified or synthetic mycolactone together with LPS, *M. ulcerans* bacteria, and mycolactone-containing extracellular vesicles (EVs) (66). This latter preparation, derived from the extracellular matrix of *M. ulcerans*, contains high concentrations of mycolactone, as well as a variety of proteins, carbohydrates and lipids (78).

The induction and biosynthesis of IL-1β differs from that of many other cytokines, chemokines and inflammatory mediators such as TNF, IL-8 and Cox-2 (**Figure 1D**). In common are the pathways that induce mRNA expression and therefore protein synthesis, such as NF-κB signaling (often referred to as “Priming” or “Signal 1”). Following *M. ulcerans*, or mycolactone-containing EV exposure, it has been demonstrated that priming of macrophages is probably *via* activation of TLR2, since genetic knockout of this receptor ablated the response (66). Although TLR production is presumably sensitive to mycolactone (47, 79), these priming signals occur very rapidly (minutes), before any depletion is likely (hours/days) (15, 16). Importantly, however, IL-1β lacks a signal peptide to direct the translating protein into the canonical secretory pathway utilizing Sec61 at the ER. Instead, IL-1β is translated as pro-IL-1β in the cytosol. A separate signaling pathway that culminates in the activation of a complex known as the inflammasome (referred to “Activation” or “Signal 2”) is responsible for cleaving pro-IL-1β into biologically active mature IL-1β. In macrophages, activation of the NLRP3 inflammasome (a complex formed of NLRP3, ASC and pro-caspase-1) cleaves and activates pro-caspase-1, which is then able to cleave pro-IL-1β, although alternative mechanisms also exist (80). Activators of the NLRP3 inflammasome are known to include microbial infection, particulate matter and ATP (81). While the precise mechanism involved in the mycolactone-dependent activation of the NLRP3 inflammasome is not known (66), it is highly likely that the toxin is providing “Signal 2”.

We now confirm that mycolactone induces IL-1β release by macrophages (**Figure 2A**). As before (66) mycolactone alone was not sufficient to induce IL-1β production, requiring LPS or intact *M. ulcerans* bacteria. However, mycolactone alone can super-induce IL-1β secretion in a well-defined *ex vivo* model of rheumatoid arthritis (RA) (67, 71–76), consisting of cultures of synovial membrane cells from RA patients who have undergone elective joint replacement surgery (**Figure 2B**). This mixed population of cells spontaneously releases a range of cytokines, chemokines and other mediators (See **Figure 2** legend for details). Within these cultures, endogenous damage associated molecular patterns (DAMPs) activate TLRs providing the priming signal to induce pro-IL-1β [reviewed in (82)]. Consequently, low amounts of mycolactone are able to super-activate Signal 2 and boost the level of IL-1β released approximately 4-fold.

Once produced, several mechanisms for secretion of IL-1β have been proposed. All of these are independent of the canonical secretory pathway and Sec61-dependent translocation of proteins into the ER, and are known as unconventional secretory pathways (83) (**Figure 1D**). The precise mechanism taking place in a particular circumstance depends on a variety of factors. A major exit route relies on cleavage of gasdermin D (GSDMD) by caspase-1 following activation of the inflammasome. The N-terminal fragment oligomerizes, forming pores in the membrane that facilitate the release of IL-1β and that can also induce pyroptosis (84). Recent evidence has argued that GSDMD-mediated IL-1β release is a tightly regulated process independent of cell death (85), and while pyroptosis is a consequence of GSDMD pore formation, it is not a prerequisite for IL-1β release (83). An alternative pathway, involving different types of cellular vesicles and/or membrane-bound organelles, can also secrete IL-1β. TMED10 was recently identified as the translocon for IL-1β into the ER–Golgi intermediate compartment (ERGIC) (86). The autophagy pathway is also implicated in regulation of IL-1β production at multiple levels, controlling inflammasome activity, promoting processing of pro-IL-1β and mediating secretion (87).

Hence, the recent discovery of IL-1β in BU lesions, and its induction by mycolactone in macrophages is entirely in line with our current knowledge of the cell biology and biochemistry of Sec61 inhibition by mycolactone.

## Discussion - Potential Implications of IL-1β Induction for Buruli Ulcer Pathogenesis

IL-1β is well known to induce inflammatory cytokines, chemokines and other mediators from a wide range of cell types (80, 88). The resultant local inflammation attracts an influx of immune cells to a site of infection, hence IL-1β release in BU could impact the ability of the host to respond to *M. ulcerans* infection, by enhancing the activation state of surrounding cells, including innate immune macrophages and neutrophils. In the mouse footpad model of *M. ulcerans* infection, treatment with the non-specific steroid anti-inflammatory dexamethasone reduced footpad swelling (66), suggesting that IL-1-driven proinflammatory responses may contribute to BU pathology. However, its potential role in controlling the infection has not yet been reported. Pre-existing IL-1β may also play a role in driving paradoxical responses that are observed at the outset of antibiotic treatment. As well as directing pathogenesis and antimicrobial immune responses, IL-1 also plays an important role in wound healing (89, 90), which likely becomes important during spontaneous healing. Moreover, other IL-1 family cytokines utilize the same strategies for protein expression and unconventional protein secretion, including IL-18, IL-33, IL-36, IL-37, and IL-38 (88, 91). Hence, these other cytokines may also bypass mycolactone’s blockade of co-translational translocation and may be present in *M. ulcerans*-infected tissues, further modulating immune responses. Indeed, IL-18 production by LPS-stimulated macrophages is increased, not blocked, by mycolactone (66).

A further downstream target of IL-1β that likely affects both pathological and protective responses to *M. ulcerans* infection is the vascular endothelium, which lines the blood vessels making up the skin microvasculature. Endothelial cells are extremely responsive to IL-1β, which modulates a range of functions, depleting junctional β-catenin and VE-cadherin (thus increasing vascular permeability) and down-regulating anticoagulant proteins such as thrombomodulin and the EPCR. IL-1β also induces procoagulant PAI-1, enhances tissue factor expression, and promotes angiogenesis *via* upregulation of adhesion molecule and vascular endothelial growth factor (VEGF) expression (91). Importantly, we have recently shown that mycolactone and IL-1β have an additive effect on both thrombomodulin depletion from endothelial cells and vascular permeability increase (12), suggesting that the local production of IL-1β may further worsen the endothelial cell dysfunction induced by mycolactone (11).

However, these downstream effects are all dependent on IL-1 receptor expression and are complicated by the fact that physiological responses to IL-1 are moderated by a complex system of receptors, co-receptors, ligands, and endogenous antagonists. IL-1β is recognised by its cognate receptor, IL-1R1, which then binds to a co-receptor IL-1R3/IL-1RAcP, triggering intracellular signalling. However, some cells, including neutrophils, express both IL-1R1 and the decoy receptor IL-1R2 (92) which increases the amount of IL-1β needed to activate them. On the other hand, highly sensitive cells, such as endothelial cells, express IL-1R1 but undetectable IL-1R2 (93, 94). A further problem in interpretation of the role of IL-1 family proteins in BU arises because the IL-1 receptors are all single-pass type I membrane proteins secreted through the conventional ER-Golgi pathway and, as such, are likely to be susceptible to mycolactone inhibition (**Figures 1C, D**) (27). Indeed, flow cytometry analysis of IL-1R1 expression by primary dermal microvascular endothelial cells shows that this receptor is susceptible to mycolactone-dependent loss (**Figure 2C**). The closer cells are to the infected tissue and the source of mycolactone production the more likely they are to be depleted of receptors and therefore insensitive to IL-1β.

In conclusion, IL-1β is likely to be a double-edged sword in BU. The question of whether it is driving the pathology, protecting against further expansion of the infection, or both, should be urgently addressed as it offers an attractive alternative approach to therapy. IL-1β targeting with canakinumab, or its cell surface receptors with Anakinra, are proposed treatments for a variety of conditions including vasculitis and diabetic foot ulcers (95–101). Similarly, diabetic mice receiving IL-1β neutralizing antibodies display reduced inflammation and enhanced re-epithelialization of skin wounds (99). However, in the context of the local inflammation, abnormal vascular phenotype and long-lasting wounds in Buruli ulcer, there are a number of issues that need to be considered. We should thoroughly examine whether the level of IL-1β and its receptor in affected tissues is sufficient to activate signaling, what happens during and after antibiotic treatment and whether its presence promotes or hinders wound healing. Understanding the role of IL-β in BU may allow us to develop new treatments that aid recovery from this devastating disease.

## Data Availability Statement

The original contributions presented in the study are included in the article/supplementary material. Further inquiries can be directed to the corresponding author.

## Ethics Statement

The studies involving human participants were reviewed and approved by Riverside Research Ethics Committee REC number: 07/H0706/81. The patients/participants provided their written informed consent to participate in this study.

## Author Contributions

BH, LT-HH, and SS performed laboratory analysis. BH and LH prepared schematics with suggestions from SS and RS. RS prepared data figures and Chimera poses. BH, LT-HH, and RS wrote the first draft of the manuscript. All authors contributed to manuscript revision, read, and approved the submitted version.

## Funding

RS holds a Wellcome Trust Investigator Award in Science (202843/Z/16/Z). This work was also supported by a grant from the Kennedy Institute Trustees.

## Conflict of Interest

The authors declare that the research was conducted in the absence of any commercial or financial relationships that could be construed as a potential conflict of interest.

## Publisher’s Note

All claims expressed in this article are solely those of the authors and do not necessarily represent those of their affiliated organizations, or those of the publisher, the editors and the reviewers. Any product that may be evaluated in this article, or claim that may be made by its manufacturer, is not guaranteed or endorsed by the publisher.
